# Sonic hedgehog (Shh)/Gli modulates the spatial organization of neuroepithelial cell proliferation in the developing chick optic tectum

**DOI:** 10.1186/1471-2202-13-117

**Published:** 2012-10-02

**Authors:** Melina Rapacioli, Joao Botelho, Gustavo Cerda, Santiago Duarte, Matías Elliot, Verónica Palma, Vladimir Flores

**Affiliations:** 1 Interdisciplinary Group in Theoretical Biology, Department Biostructural Sciences, Favaloro University, Solís 453 (1078), Buenos Aires, Argentina; 2 FONDAP Center for Genome Regulation, Faculty of Science, University of Chile, Santiago, Chile; 3 Institute of Cell Biology and Neurosciences, School of Medicine, University of Buenos Aires-CONICET, Paraguay 2155 (1121), Buenos Aires, Argentina

**Keywords:** Shh/Gli pathway, Neuroepithelial cell proliferation, Optic tectum, Non-linear signal analyses, *In ovo* cyclopamine treatment, Purmorphamine

## Abstract

**Background:**

Sonic hedgehog (Shh)/Gli pathway plays an important regulatory role on the neuroepithelial cells (NEc) proliferation in the dorsal regions of the developing vertebrate Central Nervous System. The aim of this paper was to analyze the effect of the Shh/Gli signaling pathway activation on the proliferation dynamics and/or the spatial organization of the NEc proliferation activity during early stages of the developing chick optic tectum (OT). *In ovo* pharmacological gain and loss of hedgehog function approaches were complemented with *in vivo* electroporation experiments in order to create ectopic sources of either Shh or Gli activator (GliA) proteins in the OT. NEc proliferating activity was analyzed at ED 4/4.5 by recording the spatial co-ordinates of the entire population of mitotic NEc (mNEc) located along OT dorsal-ventral sections. Several space signals (numerical sequences) were derived from the mNEc spatial co-ordinate records and analyzed by different standardized non-linear methods of signal analysis.

**Results:**

*In ovo* pharmacologic treatment with cyclopamine resulted in dramatic failure in the OT expansion while the agonist purmorphamine produced the opposite result, a huge expansion of the OT vesicle. Besides, GliA and Shh misexpressions interfere with the formation of the intertectal fissure located along the dorsal midline. This morphogenetic alteration is accompanied by an increase in the mNEc density. There is a gradient in the response of NEcs to Shh and GliA: the increase in mNEc density is maximal near the dorsal regions and decrease towards the OT-tegmental boundary. Biomathematical analyses of the signals derived from the mNEc records show that both Shh and GliA electroporations change the proliferation dynamics and the spatial organization of the mNEc as revealed by the changes in the scaling index estimated by these methods.

**Conclusions:**

The present results show that the Shh/Gli signaling pathway plays a critical role in the OT expansion and modelling. This effect is probably mediated by a differential mitogenic effect that increases the NEc proliferation and modulates the spatial organization of the NEc proliferation activity.

## Background

Sonic hedgehog (Shh) is critical for patterning, proliferation, and differentiation in a variety of tissues [1-4]. The effect of Shh is transduced by the Gli transcription factors (Gli1, Gli2 and Gli3) that have distinct and overlapping roles [5,6].

Previous studies on the roles of Shh/Gli signaling during early avian midbrain development showed that this pathway has a morphogen role in inducing ventral cell types as well as a role in stimulating cell proliferation and survival [7,8]. In this sense, Shh appears to be involved in regulation of size and shape in the developing midbrain [8]. Relatively few studies to date have analyzed the effect of the Shh/Gli pathway activation on the spatial organization of NE stem cells proliferative activity in the developing midbrain. It is known that the midbrain DV axis is established at a time when the vast majority of midbrain cells corresponds to NEcs (between stages 12–18; embryonic day (ED) 2–3); afterwards (ED3-4) a change in their adhesive properties lead to a reduction in cell mixing and to a clear structural regionalization into dorsal (alar) and ventral (basal) regions [9]. These processes are followed by an intensive proliferative activity in the alar plate [10,11] leading to a massive expansion of the tectal hemispheres between ED4-6.

The present study aims at analyzing (a) the effect of the Shh/Gli pathway activation on the NEc proliferation during the neuronogenic period, (b) its possible influence on the spatial organization of the proliferative activity and (c) its potential morphogenetic role in the developing chick OT. To accomplish this objective, we first characterized the morphogenetic effect of gain and loss of hedgehog function experiments by means of *in ovo* local pharmacological treatment with the agonist purmorphamine (Pur) or the antagonist cyclopamine (Cyc) [12]. This approach was complemented with gain of Shh/GliA function experiments through electroporation of the dorsal midbrain to create ectopic sources of either Shh or GliA proteins. Electroporation experiments were performed at embryonic days 1.5 and NEcs proliferation was analyzed at ED4 - 4.5, the time of maximal proliferating activity [13-16].

Preliminary statistical analyses on mitotic NEc (mNEc) spatial distribution in the developing OT showed that relative frequency histograms of inter-mitotic interval (I-MI) length do not fit either a homogeneous or a Gaussian probability distribution but display a slow exponential decay, i.e., high frequency of short I-MI and low frequency of long I-MI. Besides, previous structural analyses on the spatial distribution of Shh- and Gli-expressing cells in electroporated dorsal midbrains (DMBs) show that their spatial patterns are difficult to characterize due to their complex distribution, i.e., variability in size and shape of the Shh+ and Gli+ areas. For that reason, in this study the distribution of the mNEcs was analyzed by non-linear methods that have the ability to characterize complex spatial distributions [17-21].

In this study, several parameters were taken into account to evaluate the effect of Shh and GliA electroporations: (a) morpho- and histogenetic changes, (b) the global mNEc density, (c) the variability of the mNEc density along the D-V axis and (d) the scaling indexes of numerical sequences (signals) representative of the mNEcs spatial organization analyzed as a stochastic point process that occurs along the DV axis. The spatial organization was recorded by determining the spatial co-ordinates of every mNEc found in D-V sections of the midbrain. Three kind of numerical sequences: (a) Binary signals (0 = interphasic NEcs; 1 = mNE cell); (b) Inter-mitotic intervals length signals and (c) Mitotic density signals (number of mNEc/inner limiting membrane (ILM) area) were derived from the mNEcs spatial co-ordinate records and analyzed by means of several standardized non-linear methods of signal analysis [Power Spectral Density (PSD), Fano Factor (FF) and Hierarchical clustering analyses (HCA)]. All these methods have proven reliability to analyze the signal complexity and to characterize them as corresponding to different types of stochastic point processes [17-21].

## Results

### Morphogenetic effect of Shh signaling interference

**Figure 1:**
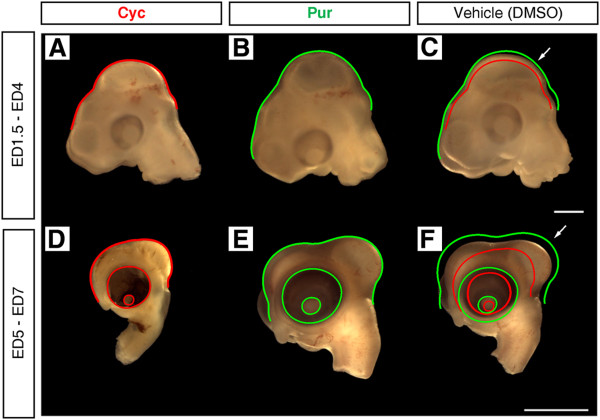
**Shh signaling is required for OT expansion.** Representative examples of the effects of pharmacological loss and gain of functions experiments with the antagonist Cyclopamine (Cyc) and the agonist Purmorphamine (Pur) applied locally in ovo at two developmental time windows. **A**-**C**. Effect on the OT morphogenesis observed at ED4 after a pharmacological treatment performed at ED1.5. Bar: 1 mm. **D**-**F**. Effect observed at ED7 after a treatment performed at ED5. Bar: 5 mm. Contours of lateral views of OT corresponding to treated embryos were delineated over the control embryos OT (arrow) in order to facilitate a direct comparison and denote the morphological differences between controls and treated embryos. Noteworthy the effect is not restricted to the OT, the dorsal forebrain (future brain hemispheres) and the optic cup (future retina) also underwent significant morphogenetic changes.

To explore whether disturbance of Shh signaling modifies the OT morphogenesis we first analyzed its phenotype after either hedgehog agonist Pur or antagonist Cyc treatments. Figure 1 illustrates the effects of gain and loss of functions experiments by means of pharmacological treatments applied locally *in ovo* over two different temporal windows (ED1.5–4.5 and ED5–7). Figure 1A-C show that ED4.5 old OT underwent significant changes in size after treatments with Cyc or Pur applied at ED1.5. Figure 1D-F illustrate the effect produced by treatments performed at ED5 and analyzed at ED7. Importantly, during both temporal windows Shh antagonist produces significant reductions in the OT, dorsal prosencephalon (future brain hemispheres) and optic vesicle (future retina) while the Shh agonist produces the opposite effects, i.e. larger than usual OT, dorsal prosencephalon and optic vesicles.

**Figure 2:**
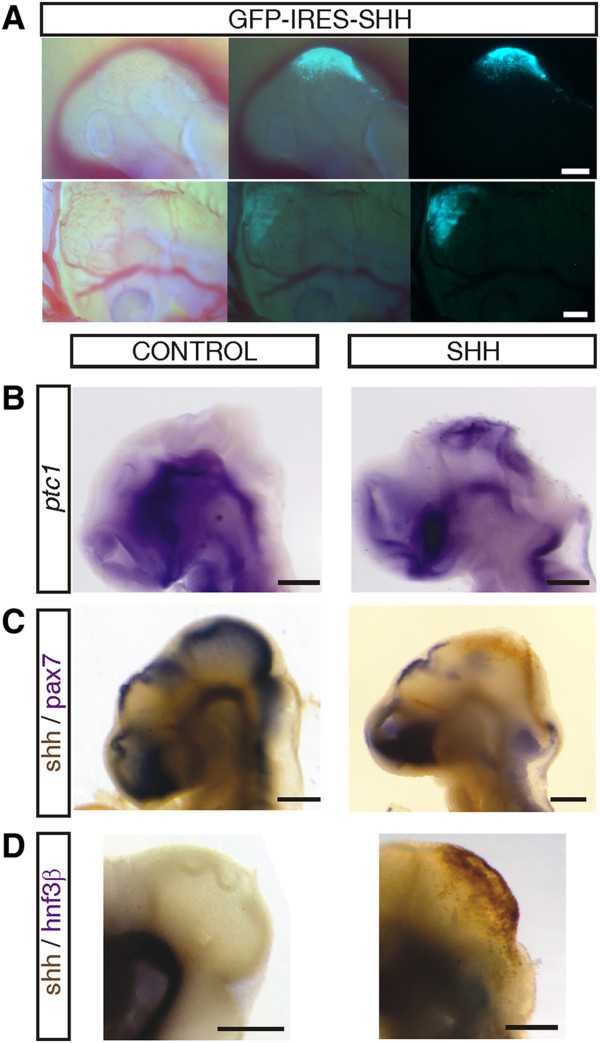
**Results of electroporation. A**. Left lateral view of the cephalic region of Stg 17 (upper panel) and Stg 21 (bottom panel) embryos electroporated at Stg 11. Patches of GFP+ areas are observed over the DMB surface. Bars: 100 μm. **B**. In situ hybridization for detection of *ptc1* expression. In the control embryo *ptc1* expression is restricted to the ventral neural tube. In the Shh electroporated embryo ectopic *ptc1* expression is observed in the dorsal midbrain. Bar: 100 μm. **C**. Double staining for Shh and Pax7. Control embryo display Shh immunoreactivity in the basal midbrain and Pax7 reactivity in the dorsal midbrain. The dorsal midbrain of the Shh electroporated embryo displays ectopic expression of Shh and downregulation of Pax7. **D**. Double staining for Shh and Hnf3β. In control specimens the ventral marker Hnf3β, typically stain the basal midbrain. In the Shh electroporated embryo the area of Shh misexpression is restricted to the dorsal; the domain of Hnf3β expression expanded laterally into a region that normally corresponds to the OT.

In order to specifically analyze the effect of Shh signalling on the developing OT, localized electroporation of exogenous Shh and GliA was performed at the DMB. Figure 2 shows the result of the electroporation procedure; patches of GFP labeling can be seen distributed over the DMB (Figure 2A). Histological examination of these GFP+ areas is illustrated in next section. To further evaluate the effectiveness of electroporation and to ascertain whether Shh related genes expressions are modified, the expression of *ptc1*, and Pax7 was analyzed (by in situ hybridization and immunohistochemistry respectively). We also analyzed the expression of Hnf3β, a typical marker of the basal midbrain.

Shh electroporated DMBs at ED4.5 displayed increased expression of *ptc1* (Figure 2B) and downregulation of pax7 (Figure 2C) in comparison to the control ones confirming that, indeed, cells located at the dorsal midbrain actively respond to Shh electroporation.

Figure 2B illustrates in situ hybridization for detection of *ptc1* expression. In the control embryo *ptc1* expression is restricted to the ventral neural tube; in Shh electroporated embryos, ectopic *ptc1* expression is observed in the dorsal midbrain. Figure 2C illustrates double staining for Shh and Pax7. Control embryos display Shh immunoreactivity in the basal midbrain and Pax7 reactivity in the dorsal midbrain. By contrast the dorsal midbrains of Shh electroporated embryos show ectopic overexpression of Shh and downregulation of Pax7.

**Figure 3:**
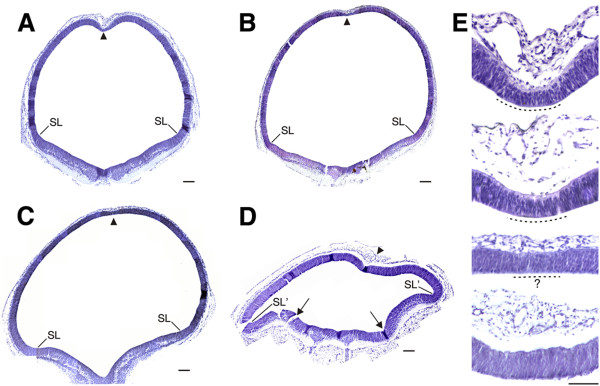
**Micrographs of D-V sections of ED4.5 midbrains (H-E stained). A**. Control. **B**. GliA electroporated. **C**. Shh non-ventralized electroporated. **D**. Shh partially ventralized electroporated, The intertectal sulcus (arrowhead) that separates the left and right tectal hemispheres tend to disappear in GliA electroporation and disappears in Shh electroporated specimens. **E**. Detail of the roof plate (the thinner neuroepithelial band at the bottom of the intertectal sulcus) alterations produced by GliA and Shh electroporations. From top to bottom: Control, GliA electroporated, Shh (non-ventralized) electroporated and Shh (partially ventralized) electroporated dorsal midbrains. Graded alterations of the roof plate (medial zones indicated with dotted lines) can be observed in electroporated specimens. In GliA treated the roof plate can still be recognized at the bottom of the intertectal sulcus; in Shh (non-ventralized) specimens the roof plate is poorly demarcated and difficult to identify; in Shh (partially ventralized) midbrains the roof plate is no longer recognized. Arrows: indicate the position of the original sulcus limitans in a partially ventralized midbrain. SL: sulcus limitans. SL’: additional sulcus limitans typical of partially ventralized specimens. Scale bars: 50 μm.

Figure 2D shows double staining for Shh and Hnf3β. In control specimens the dorsal midbrain is negative for Shh labeling and the ventral marker Hnf3β, typically stain the basal midbrain. In Shh electroporated midbrains Shh labeling is visualized dorsally and, in most cases, Hnf3β is restricted to the basal plate. This result suggest that in most cases dorsal progenitors could not be re-specified to ventral lineages, rather they are destined to differentiate toward a tectal fate even in the presence of Shh misexpression. In some Shh electroporated specimens the ventral domain of Hnf3β expression expands laterally into a region that normally corresponds to the OT. This lateral expansion of the Hnf3β+ area invading a zone that normally differentiate into OT suggest an evolution towards a basal fate and could correspond to specimens that, under histological examination, reveal a partial ventralization of the lateral region of the alar plate (see Figure 3D).

Figure 3A-D show images of D-V sections, located halfway between the cephalic and caudal ends of the MB, obtained from ED4.5 embryos. DMB submitted to GliA electroporation retained its alar character and differentiated into typical OT (Figure 3B). Shh electroporation produced two types of results: the majority (7/10) of DMBs retained their alar character and differentiated into OT (Figure 3C) but some of them (3/10) underwent partial ventralization. In this last case, the DMBs had two different regions (Figure 3D): the dorsalmost region retained the alar phenotype (“tectal region of ventralized DMB)”- while its ventral zone changed its developmental fate and differentiated into basal structures (“ventralized region of the DMB”).

**Figure 4:**
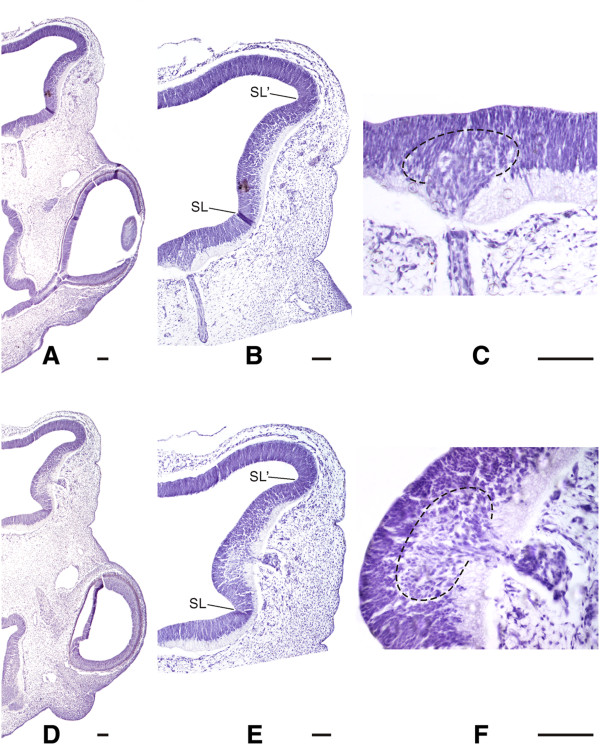
**Dorsal**-**ventral sections of a partially ventralized dorsal midbrain.** A partially ventralized dorsal midbrain typically displays a dorsal region with an alar structure and a ventralized region with a basal-like organization. **A**-**C**. These sections show the histological organization of the ventral midbrain, the ventral-medial position of the normal oculomotor nucleus (encircled by a dashed line) and the emergence of the eutopic III cranial nerve. **D**-**F**. These sections show the histological organization of the ventralized region of the dorsal midbrain, the abnormal (lateral) position of a duplicated motor-like nucleus (encircled by a dashed line), and the lateral emergence of an ectopic supernumerary cranial nerve. The original sulcus limitans (SL) can still be recognized between the ventral midbrain and the ventralized region of the dorsal midbrain. A new boundary, an additional sulcus limitans (SL’), separates the ventralized region of the dorsal midbrain from the tectal region. Scale bars: 50 μm.

Apart from their differences in size, by ED4.5 the control OT typically exhibits the incipient dorsal-medial intertectal sulcus (between the left and right hemispheres) and the roof plate at the bottom of the medial sulcus. The roof plate is identified as the thin neuroepithelial band at the dorsal midline (Figure 3A and E). Normally, the tectal roof plate later differentiates into the lamina commissurales (dorsal intertectal commissure). In GliA electroporated OT the intertectal sulcus tends to disappear but the roof plate can still be recognized in 100% of the embryos as a thinner dorsal-medial neuroepithelial band (Figure 3B and E). In Shh electroporated specimens the intertectal sulcus disappears and the dorsal aspect of both the left and the right hemitectum form a single dorsal dome without any separation between them (Figure 3C and E). In Shh electroporated (non-ventralized) DMBs the roof plate does not completely disappear; patches of poorly demarcated zones with a roof plate-like organization can be observed along the dorsal midline (Figure 3E). In partially ventralized DMB the roof plate disappeared and the dorsal midline can only be identified by the position of the dorsal medial blood plexus (Figure 3D and E). The ventralized region of the DMB changes its typical tectal structure to a histological organization characteristic of the basal region (Figure 4A and D). It is composed of groups of neurons resembling the basal organization. The neurons of these motor-like nuclei originate axon fascicles that emerge through the ventral-lateral aspects of the MB forming one or more supernumerary motor-like nerves as judged by their position (Figure 4E and F). The persistence of the sulcus limitans allows distinguishing the original basal region from the ventralized region of the DMB. An additional sulcus limitans appears between the tectal and the ventralized region of the DMB.

### Histogenetic and immunocitochemical characterization of the OT developmental stages in Shh and GliA electroporated dorsal midbrain

**Figure 5:**
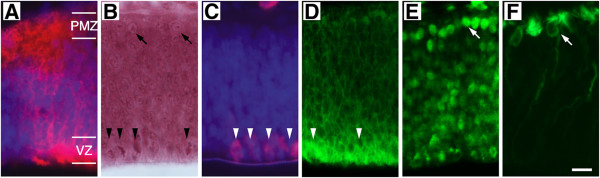
**Radial organization and immunocytochemical patterns at the end of DS1. A**. Ectopic expression of pShhiEGFP in an electroporated OT (ED4.5); patches of positive NE cell bodies at the ventricular zone (VZ) and of postmitotic neurons at the premigratory zone (PMZ) can be observed. **B**. Hematoxylin-Eosin staining. Arrowheads point to mNE cells located along ventricular zone; they show PH3 nuclear labeling (**C**) and Notch reactive cytoplasm (**D**). Arrows point to neurons in the premigratory zone; they display nuclear NeuroD reactivity (**E**) and βIIITub reactive perikarya (**F**). Bar: 10 μm.

The ventralized region of the DMB displays an altered histogenetic organization that can not be characterized within the reference provided by table of developmental stages given in Reference [11]. For that reason, only observations made in non-ventralized DMBs, i.e., DMBs that retain their tectal character, will be described in this section. Figure 5A–F show that by ED4/4.5, the OT is at the end of the developmental stage 1 (DS1). At the beginning of DS1 (ED2) the OT structure corresponds to a single layered neuroepithelium. Between ED2–ED4/4.5 the OT evolves through DS1: (a) the 1^st^ neuronal cohort (future Mes5 sensory neurons of the mesencephalic trigeminal nucleus) appears along the dorsal midline between ED2-ED4 and (b) the 2^nd^ cohort (future large efferent neurons of the SGC and SGP) appears over the entire OT neuroepithelium from ED3.5-ED4 onwards. These latter neurons are born at the ventricular zone (VZ), near the inner limiting membrane (ILM), and then move to the outermost subpial zone forming an incipient premigratory zone (PMZ).

Figure 5A show that both NEcs -ventricular zone- as well as postmitotic neurons - premigratory zone- express Shh revealing a successful electroporation procedure (Figure 5A). During this period the VZ displays the intense Notch reactivity typical of mNE cells (Figure 5D) and both the hematoxylin-eosin staining (Figure 5B) and the Phospho-histone H3 (PH3) immunolabeling (Figure 5C) reveal groups of mNEcs overlying the ILM. Besides, the NeuroD immunolabeling (Figure 5E) shows that the PMZ is populated by newly-born neurons characterized by intense NeuroD nuclear reactivity. The beta III Tubulin (βIIITub) labeling (Figure 5F) shows that these NeuroD+ neurons have already begun the early differentiating phase. These newly born neurons correspond to future large efferent neurons of the SGC. Figure 5B and C show that both the H-E staining and the PH3 immunolabeling permit easy identification of mNEcs and allow reliable recordings of their position along the D-V section (See also “Mitotic NE cell records” in Methods).

### 2D representation of mNEc records along the D-V axis

**Figure 6:**
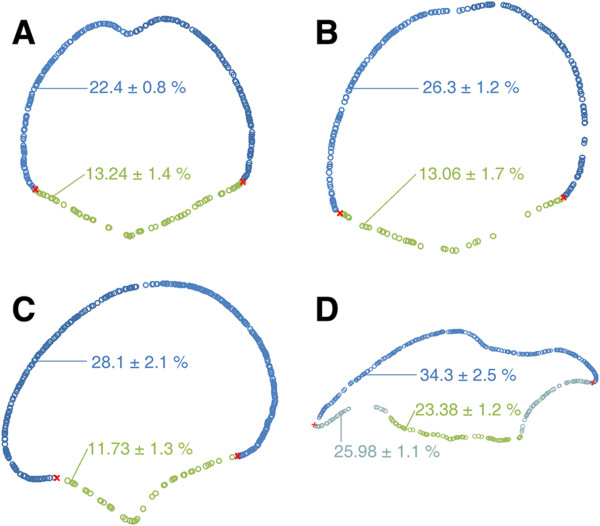
**Two dimensional representations of mNEc records. A**. Control. **B**. GliA electroporated. **C**. Shh electroporated, non-ventralized. **D**. Shh electroporated, partially ventralized. The position of each circle is specified by the spatial co-ordinates of each mNEc. Blue circles: mNEcs located along the alar plate. Green circles: mNEcs located along the ventral midbrain. Light-blue circles: mNEcs located along the ventralized region of the dorsal midline. The percentage (mean ± SD) of the ILM area occupied by mNEcs is indicated in each case. There are statistically significant differences (p<0.03) between control (**A**) vs. GliA (**B**) and Shh (**C** and **D**). The ventralized region of the dorsal midbrain and the ventral midbrain of partially ventralized dorsal midbrains (**D**) statistically differs from the other specimens (p<0.001).

Figure 6A-D correspond to 2D maps of mNEcs records obtained from control, GliA electoporated and Shh electroporated DMBs (non-ventralized and partially ventralized DMBs). The 2D maps of mNEcs allocations strictly coincide with the ILM contours indicating that the allocation of the entire population of mNEcs reliably reproduces their positions along the D-V axis (Compare with Figure 3A-D). In each case, the percentage of ILM area occupied by mNEcs is indicated. A statistical comparison shows that GliA and Shh electroporation significantly increased the area of ILM occupied by mNEcs in the OT. The increase in this parameter is even higher in the tectal region of the partially ventralized DMB.

There were not significant differences amongst the VMB of control, GliA and Shh electroporation without ventralization (Figure 6A, B and C). However, both the ventralized region of DMB and the VMB of these specimens (Figure 6D) display percentages of ILM area occupied by mNEcs significantly higher than the other specimens. It can be noted that the ventralized region of the DMB differs significantly from the tectal region of the same DMB but closely coincide with the values of the VMB.

### Statistical analyses of signals derived from mNEc records

**Figure 7:**
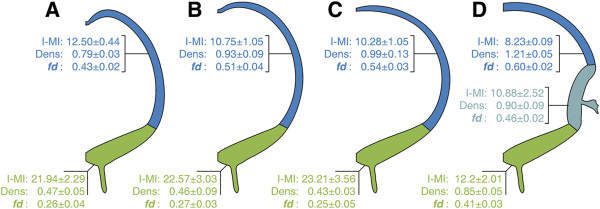
**Summary of the effects of GliA and Shh electroporations A. Control. B. GliA electroporated. C. Shh electroporated (non-ventralized). D**. Shh electroporated (partially ventralized). Values of Mean ± standard deviation of inter-mitotic interval length (I-MI), mitotic NEcs density (Dens) and fractal dimension (*fd*), are indicated for optic tectum (blue), ventral midbrain (green) and ventralized dorsal midbrain (light blue). These values correspond to global means calculated over the entire D-V axis of each region. See descriptions in the text.

Figure 7 summarizes the effects of GliA and Shh electroporation on the I-MI length, the mNEc density and the fractal dimension (estimated by the space filling property). This figure compares the values of these parameters measured in different regions of the MB in control specimens, GliaA electroporated and Shh electroporated MBs (non-ventralized and partially ventralized DMBs). These values correspond to global estimations performed over the entire D-V axis of each region.

The global mean I-MI length was significantly shorter in GliA and Shh electroporated (non-ventralized) DMB than in the control OT indicating closer positions between neighboring mNEcs (Figure 7A-C). There were no significant differences amongst the VMB of these three specimens. The effect of Shh was more intense in specimens with partially ventralized DMB (Figure 7D). In this case, the mean I-MI length in the tectal region was significantly lower than the value corresponding to the OT of the other three specimens. The value corresponding to the ventralized region of the DMB was higher than that of the tectal regions of the same specimens and approximate to that of the VMB of the same specimens. Besides, the VMB of these specimens underwent a significant and remarkable decrease in I-MI length compared with the VMB of the other three specimens.

With regards to the mNEc density in the alar plate, GliA and Shh electroporation results in significant increases of this parameter with respect to the control OT. However, no significant changes were detected amongst the VMBs of the three specimens. In Shh electroporated partially ventralized DMB, the mNEc density in the tectal region was significantly higher than those of the corresponding regions of the OT of the other three speciments. The ventralized region of the DMB underwent a decrease in mNEc with respect to the tectal region of the same specimens and also with respect to the alar plate of Shh electroporated specimens without ventralization. Interestingly, the mNEc density in the ventralized region of the DMB and in the VMB of the same specimens closely coincide. These mNEc densities however, differs significantly from the values observed in the VMB of the other three specimens.

Consistently with the increase in the global mNEc density, the Box Counting method applied to binary signals revealed a significant increase in the fractal dimension (space filling property) in GliA and Shh electroporated (non-ventralized) DMB (Figure 6A-C). In specimens with a partially ventralized DMB the value of the fractal dimension also significantly differed from the other three specimens.

All these analyses show that the pattern of values describing the proliferative behavior in the ventralized region of the DMB closely coincides with that of the VMB of the same specimens. These results indicate that the ventralization of the DMB does not only involve a change in its histological organization but also a change in the proliferative behavior of the NEc population residing in the ventralized region.

**Figure 8:**
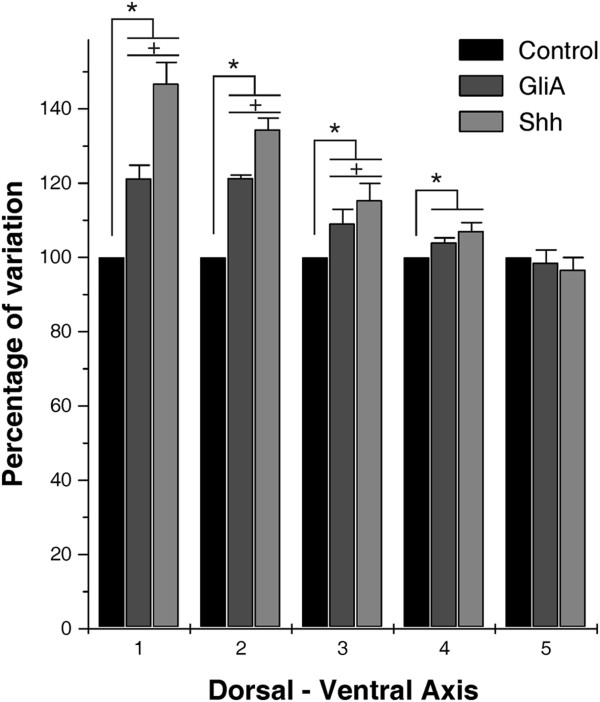
**Space-dependent differences of Shh and GliA effects along the D-V axis.** Each bar represents the mean ± standard deviation of the mNEc density measured in 500 μm length spatial windows located at defined positions along the D-V axis. The means mNEc density measured in controls OT were taken as reference (100%). A decreasing responsiveness to Shh and GliA can be observed from the dorsal region (1) to the ventral one (5). 1: dorsal region, 3: halfway between the OT dorsal and ventral zone; 5: OT-tegmental boundary. 2 and 4 represents intermediate positions between 1 and 3 and between 3 and 5 respectively. *: indicate statistically significant differences (p<0.001) measured by the Z test. +: indicate statistically significant differences (p<0.01) between GliA and Shh.

Analyses of local variations in mNEcs density performed in GliaA electroporated and in Shh electroporated without alar plate ventralization, estimated in successive 25 μm length windows along the D-V axis, indicate the existence of graded space-dependent differences in response to electroporation along this axis (Figure 8). In fact, the change in mNEc density in response to the GliA and Shh electroporation is maximal near the dorsal midline and from this zone decreases gradually towards the OT-VMB boundary. These analyses also show that the response to Shh electroporation is higher than the response to GliA electroporation.

### Non-linear analyses of signals derived from mNEc records

**Figure 9:**
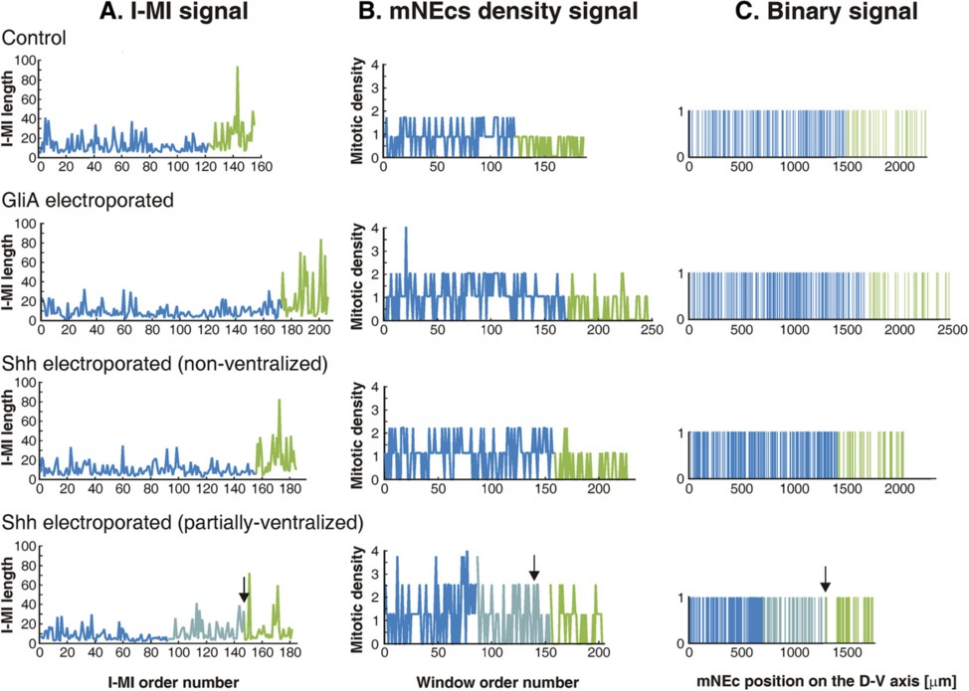
**Signals derived from the mNEc records. A**. I-MI signals. Each value corresponds to the distance between two adjacent mNEcs. **B**. Mitotic density signals. Each value corresponds to the density of mNEcs (number of mNEcs/100 μm^2^) in successive spatial windows along the D-V axis (window length = mean I-MI length). **C**. Binary signals. Represent the mNEcs distribution as stochastic point process. “***1*** *s*”: indicate the position of the centre of each mNEcs along the D-V axis; “***0*** *s*”: represents the absence of mNEcs. In all cases the D-V axis position coincides with the abscissa and the dorsal midline corresponds to the position “0” of the “x” axis. The blue subseries correspond to the alar plate (OT), light blue subseries correspond to the ventralized region of the dorsal midbrain and the green subseries to the ventral midbrain. Arrows: indicate the original dorsal/ventral midbrain boundary.

Figure 9A-C illustrate examples of I-MI signals, mNEc density signals and binary signals derived from mNEc records performed in control OT, GliA electroporated DMB and Shh electroporated (non-ventralized and partially ventralized) DMBs.

#### Power spectral density (PSD)

**Figure 10:**
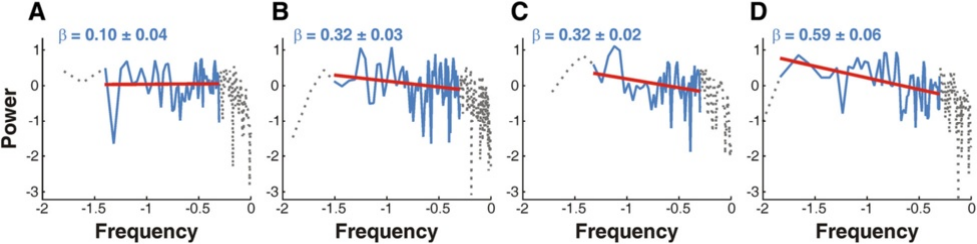
**Power spectral density (PSD) and estimation of the scaling index β.** Log-log plots of the PSD of mNEc density signals obtained from controls (**A**) and GliA electroporated (**B**) and Shh non-ventralized (**C**) and tectal region of the partially ventralized Shh electroporated dorsal midbrains (**D**). Means values of β (mean ± SD) are indicated. The slopes of the lines (red lines) fitted by least squares linear regression is – β.

Figure 10A-D illustrate results of classical spectral analyses performed on mNEc density signals corresponding to controls and GliA and Shh electroporated alar plates. In the case of Shh electroporations with partially ventralized DMB, only the alar (non-ventralized) region was included in this analysis. Values of the means and the standard deviations of the scaling index β obtained from the slope of the lines in the log-log plots are indicated. The statistical analysis of the differences amongst the means values of β indicates the existence of significant differences between the control OT and the remaining alar plates (p<0.05, p<0.01 and p<0.001 respectively). The value of β estimated in the tectal region of the partially ventralized DMB was significantly higher that the values obtained in GliA and Shh electroporated OT. These results indicate that both exogenous GliA and Shh modified the mNEcs spatial organization and that the ventralized specimens underwent a more drastic reorganization of mNEcs.

#### Fano factor (FF)

**Figure 11:**
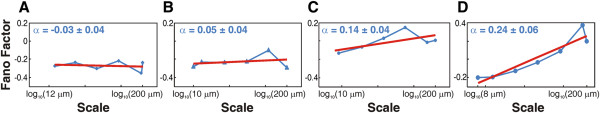
**Fano Factor (FF) analyses of binary signals.** Log-log plot of the FF algorithm applied to binary signals obtained from control (**A**), GliA electroporated (**B**) and Shh non-ventralized (**C**) and tectal region of Shh partially ventralized electroporated dorsal midbrains (**D**). Means values of α (mean ± SD) are indicated in each case. α = the slope of the line estimated by least squares linear regression.

Figure 11A-D illustrate results of FF analyses applied to binary signals corresponding to control, GliA and Shh electroporated alar plates. The values of the mean and the standard deviation of the scaling index α obtained from the slope of the line in the log-log plots are indicated in each case. The statistical comparison amongst the values of α indicates the existence of significant differences between control vs. Shh electroporated specimens (p<0.04). These last values are also significantly higher than the one corresponding to the GliA electroporated alar plates. The values of α obtained from controls OT approximate the value corresponding to standard Poisson-like process while those of the Shh treated OT correspond to stationary correlated stochastic point process. Consistently with the preceding analysis, the value α estimated in the tectal region of the partially ventralized DMB was significantly higher than the value of the OT of the non-ventralized DMB.

#### Hierarchical clustering analysis (HCA)

**Figure 12:**
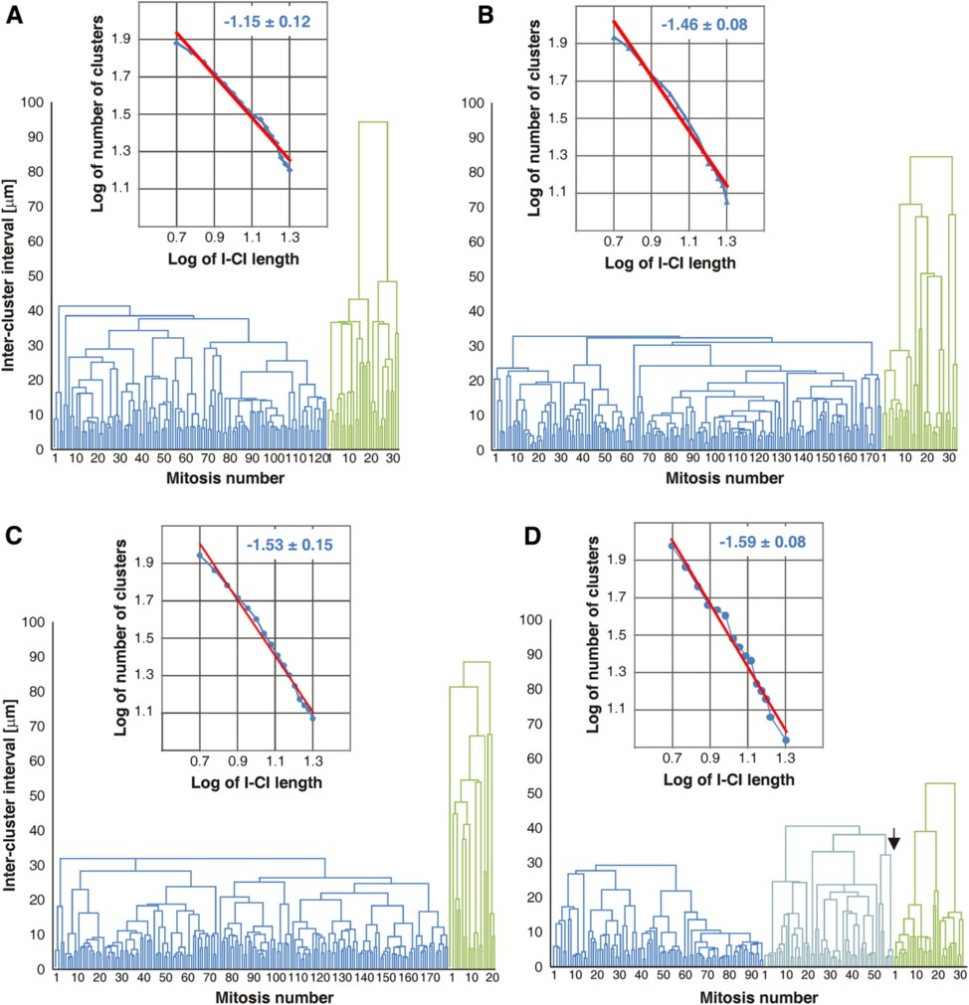
**Hierarchical clustering analyses (HCA) of binary signals.** Dendrograms obtained by means of a HCA of the binary signals and log-log plots of the number of clusters as a function of the inter-cluster interval length obtained from controls (**A**) and GliA electroporated (**B**) and Shh non-ventralized (**C**) and Shh partially ventralized (**D**) electroporated midbrains. The dendrogram reveals that mNEcs are organized as clusters of different hierarchies (clusters within clusters). The height of the connectors (Π) indicates the level of aggregation, i.e., the scales at which two clusters of a given hierarchy aggregates into a single cluster of a higher hierarchy. The log-log plots show a power law behavior revealing the absence of a typical mean cluster size and that mNEcs are distributed in a continuous hierarchical set of clusters within clusters across scales.

Figure 12A-D show dendrograms obtained by means of hierarchical clustering analyses of binary signals corresponding to control, GliA electroporated and Shh electroporated (non-ventralized and partially ventralized) DMBs respectively. Dendrograms corresponding to the four different conditions display obvious visual differences. These differences can be quantitatively evaluated by the values of the slope of the lines in the log-log plots of the number of clusters as a function of the inter-cluster interval length.

The values of the scaling indexes estimated in GliA and Shh electroporated alar plates were significantly higher that the one corresponding to the control OT. Besides, coinciding with preceding analyses, the scaling index estimated in the tectal region of the partially ventralized DMB was higher than the value observed in GliA and Shh (non-ventralized) eletroporated alar plates. This analysis confirms that GliA and Shh electroporations significantly modify the power law that governs the spatial organization of the mNEc and that this effect is higher in Shh electroporated alar plates, especially in the tectal region of the partially ventralized DMBs.

## Discussion

### Morphogenetic effect of Shh and Gli electroporation

It is known that the midbrain D-V polarity is determined, i.e., irreversibly established, during the early stages [HH Stg 12–18 (ED 2–3)], onto a relatively small pool of progenitor NE cells. Afterwards, by means of an intensive proliferative activity, these alar- or basal-committed NEcs give rise to populations of alar-derived or basal-derived neurons, respectively [9]. According to previous studies the D-V axis commitment takes place during HH Stg16, a brief period of only 5–6 hs. Given that the biological activity of the Shh protein peaks around 20–24 h after electroporation and considering that the effect persists for at least 48 h it is quite plausible that in our experiments, apart from their mitogenic influence, GliA and Shh proteins could be actively influencing the midbrain DV patterning. Given the short time period along which the axis is determined, small differences in the developmental stage of the embryos (a difference of only 5 h) could lead to distinct results. This could explain the existence of two different kinds of results in response to Shh electroporation. In our opinion, Shh electroporations that did not result in DMB ventralization correspond to embryos whose midbrain D-V axis were already fixed at the time when the Shh protein was overexpressed. Given that the process of D-V axis determination progresses from the dorsal to the ventral regions, it is plausible that in some cases the overexpression of the Shh protein occurs at a time when the axis was already fixed in the dorsal region but it was still plastic in the ventral region of the DMB. This could explain why the partially ventralization in all cases involved only the ventral zone of the alar plate and not the dorsal one.

This study shows that *in ovo* pharmacological injections of agonist and antagonist of the Shh pathways have profound effect on the OT, dorsal forebrain and optic vesicle. While cyclopamine has been shown to inhibit Shh signaling in the chick embryo, to our knowledge, this is the first demonstration of an *in vivo* effect of the hedgehog agonist purmorphamine.

The present work shows that, in specimens that did not undergo DMB ventralization, Shh and GliA proteins display a mitogenic effect, specifically localized at the alar plate. In these specimens, nor GliA neither Shh modify the proliferative activity at the VMB. This study also shows that, in specimens who’s DMB underwent partial ventralization, the Shh protein exerts both a mitogenic and a morphogen-like effect: (a) The morphogen-like effect is revealed by the change in the developmental fate of the ventral zone of the alar plate. The ventralization of the alar plate does not only involve a change in its evolution towards a basal-like histogenetic process but also a change in the pattern of NEcs proliferation. In fact, the ventralized zone of the DMB undergoes a decrease in proliferation, with respect to the tectal region of the same and other specimens, and acquired a pattern of proliferation that closely resembles the proliferative behavior of the NEcs located in the basal plate of the same specimens. This result is consistent with previous reports demonstrating that ectopic Shh changed the fate of the mesencephalic alar plate to that of the basal plate, and decrease the massive cell proliferation that normally occurs in the developing tectum [22]; (b) the mitogenic effect, in these specimens, is revealed by the fact that (1) the tectal region of the partially ventralized DMB displays a higher proliferation than the corresponding regions of the alar plate of the others specimens and (**2**) the ventralized region of the DMB and the VMB display a higher proliferation than the VMB of the other specimens.

Shh and GliA proteins significantly modify the proliferative behavior of the DMB mNEc and this effect is accompanied by a morphogenetic anomaly at the OT dorsal midline. GliA and Shh electroporation alter the intertectal sulcus formation. It is considered that the medial-dorsal furrow, i.e., the longitudinal groove preceding the intertectal fissure (intertectal sulcus) formation, involves a relative shortening of the dorsal midline -with respect to the lateral region of each hemitectum- due to a relative decrease in the NEc proliferation along the dorsal-medial region [10]. The dorsal-medial groove formation is a relevant morphogenetic feature of the DMB since it is required for intectectal sulcus formation and subsequent segregation of the right and left tectal hemispheres. This process is normally followed by the differentiation of the lamina commissuralis and the inter-tectal commissure at the bottom of the sulcus. Both GliA and Shh electroporation of the DMB, in different degrees, interfere with this morphogenetic event. In fact, GliA electroporation reduces the furrow depth and Shh electroporation completely impedes the medial fissure formation leading to a severe failure in the segregation of the right and left tectal hemispheres. In specimens with partially ventralized DMB Shh electroporation lead to a complete absence of the roof plate.

It is interesting that the pharmacological treatment with the Shh agonist purmorphamine also produces a lengthening of the OT dorsal midline resulting in an abnormally exaggerated dorsal convexity (V. Palma unpublished results).

### Statistical analyses of the effect of Shh/GliA pathway on the NE cell proliferation. A graded responsiveness to GliA and Shh electroporation

The above-described structural anomaly is accompanied by alterations in several quantitative parameters of proliferation activity. In specimens with non-ventralized DMB, the increase in mNEc density takes place specifically at the OT; the mNEc density at the VMB remains unchanged.

It is interesting that the effect of GliA and Shh electroporations is not homogeneous over the entire alar plate. Analyses of local variations in mNEc density performed along the D-V axis showed that the effect of GliA and Shh elecroporations on this parameter is space-dependent. In both cases the increase in mNEc density is maximal near the dorsal midline and, from this zone, the density decreases down to the OT-DMB boundary where the difference completely disappears. This boundary is easily recognizable by the persistence of the sulculs limitans and by their histological differences. This result suggests the existence of a D-V gradient in the sensitivity (or competence) of the NEc to proliferate in response to the GliA or Shh proteins.

The multiple roles of Shh signaling during the development of CNS depend on the region, timing, and concentration of Shh expression [1-8,23-26]. For instance, it has been shown that the Shh expression level in the dorsal telencephalon is extremely low as compared with that in the ventral telencephalon. The ‘high level’ of endogenous Shh expressed in the telencephalic medial ganglionic eminence is important for ventral patterning and differentiation of GABAergic interneurons. The ‘low level’ of endogenous Shh in the dorsal telencephalon plays important roles in the proliferation, differentiation, and positioning of cortical neurons through fine-tuning of cell cycle kinetics [27].

### Non-linear analyses

The signals derived from the mNEc records are considered as realizations of stochastic point processes. They were mathematically analyzed to determine whether they correspond to non-correlated stochastic processes (white noise) or correspond to correlated stochastic processes with memory. These analyses consistently show that both Shh and GliA electroporations modify the scaling indexes of the signals indicating changes in the proliferation dynamics and/or the spatial organization of mNEcs.

The values of the index of space filling property estimated by the box counting method significantly differ comparing normal versus electroporated DMBs. The change in the scaling index β estimated by the PSD of the mNEc density signal indicates that Shh and GliA electroporations change the spatial arrangement of mNEcs. In fact, the significant increase in the value of β observed in GliA and Shh electroporated DMB indicates that these signals display a higher correlation than those obtained from the controls DMBs. The signals obtained from Shh and GliA treated specimens behave as stationary correlated stochastic processes with memory or space-dependency being the correlation higher in the Shh treated specimens than in the GliA treated ones. These results reveal changes in the spatial organization of the mNEcs and suggest that the increase in concentration of exogenous Shh and GliA proteins have a synchronizing influence on the NEcs proliferation.

The change in the scaling index α estimated by the FF analyses of the binary signals also reveals differences between controls and Shh treated specimens. In fact, the values of α ≈ 0.0 that characterize the signals recorded from control specimens correspond to homogeneous Poisson-like processes. By contrast, the values of α > 0.0 estimated from the binary signals obtained from Shh treated midbrains reveal that they behave as correlated stochastic point process with self-similarity (fractality). The FF algorithm was not able to reveal significant differences between controls and GliA treated midbrains. It is interesting that these analyses reveal an even higher correlating influence of Shh on mNEc density signals derived from specimens with partially ventralized DMB.

With regards to the HCA, both the GliA and the Shh electroporations increase the slope of the log-log plot of the number of clusters as a function of the inter-cluster interval length; this increase is higher in Shh treated specimens. It is interesting to spatially interpret the increase in the slope observed in this analysis. The increase in the scaling index takes place over a range of scales ranging from 5 to 20 μm. It must be noted that at ED4.5 only 22% of the ILM area is occupied by mNEcs and that GliA and Shh increase that percentage to only 26% and 29%, respectively. This implies that, normally, there is a large area of the ILM, i.e. around 80% of the total area, occupied by interphasic cells. Given that small clusters of mNEcs are mainly separated by short inter-cluster intervals and that large clusters are separated by long inter-cluster intervals, the addition of “new” mNEcs produced by Shh should mainly occur, by chance, at the longer intervals. Surprisingly, the increase in the slope observed over the range of scales 5 to 20 μm reveals that the intercalation of additional mNEcs does not takes place at random but, preferentially, at the short intervals, i.e. between or within the smaller clusters.

**Figure 13:**
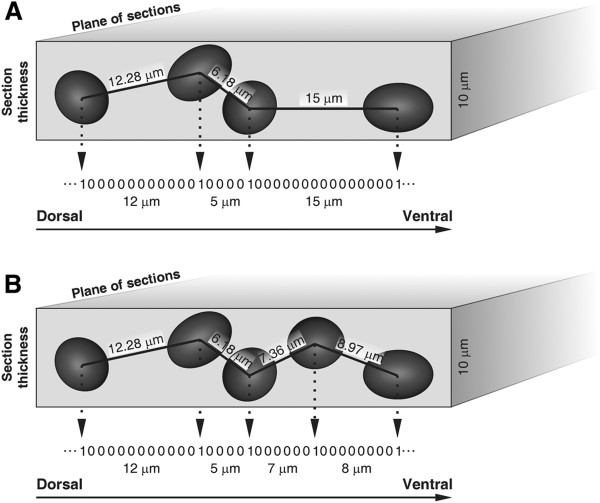
**Schematic representation of mitotic neuroepithelial cells intercalation among adjacent mNECs.** Schematic representation of a planar view of the ILM where “x” represents the D-V axis and “y” corresponds to the section thickness. Binary signals correspond to the projection of the distance of adjacent mNEcs over the ILM area.

It must be mentioned that the smallest distance between two adjacent mNEcs equals the mean cell diameter (around 6 μm). Thus, I-MI or inter-cluster intervals smaller that 6 μm corresponds to adjacent mNEc that are partially overlapped within the 10 μm thick histological section. Figure 13 shows how these mNEc are partially overlapped with respect to the line (representing the ILM) along which the I-MI intervals are measured. Thus, most of the additional mNEc produced by GliA or Shh electroporation appeared adjacent to another mNEc. Considering that the increase in the mNEc density and the intercalation of additional mNEcs occurs preferentially between or within the smaller clusters [10], it is likely that the proliferative effect of the Shh pathway preferentially takes places at zones where mNEc are involved in symmetrical divisions and neuroepithelial tangential expansion.

The Shh effect is quantitatively higher than the GliA effect. Values corresponding to GliA treated midbrains were consistently intermediate between those corresponding to controls and Shh treated midbrains. The fact that Shh more efficiently stimulate NEc proliferation and installs a higher coordination on the proliferative activity suggests that Shh may have other effectors besides GliA. Of note, our previous studies suggested a non-canonical role of Shh modulating EGF mitogenic responsiveness when we showed that this factor regulates the expression of EGFR in neural stem cells in the dorsal brain [28,29]. At this point we cannot rule out a crosstalk with other important mediators of OT growth such as BMPs and canonical Wnt signaling.

### On the role of Shh and Gli during the tectal corticogenesis

Shh acts as a morphogen in the ventral patterning of the CNS [27,28]. A high-ventral to low-dorsal gradient of Shh from the notochord and amplified by the floorplate controls the spatial organization of the different neuronal type determination in the spinal cord, hindbrain and midbrain basal plates [8,30]. Shh, secreted by the prechordal mesoderm, also participates in the patterning of the ventral diencephalon, the medial region of the forebrain [31]. The Gli transcription factors, downstream effectors of Shh signaling, are also involved in this spatially organized process of Shh signaling [5,6,23].

Apart from its role in the ventral regions of the CNS, the Shh pathway also has an important histogenetic function by regulating neuronogenesis in the associative areas derived from the alar plates of the brain [4,32]. Early differentiated Purkinje neurons release Shh and stimulate granule neurons progenitors to proliferate [33,34]. In this way a macroneuron population regulates the number of associative microneurons and then the amount of afferent axons onto its own dendritic tree.

Hedgehog signaling is known to accelerate progression through the cell cycle in many model systems. Shh is involved in development of the dorsal neocortex, diencephalon and midbrain [5,35]. A Shh-dependent signaling relay has been proposed to regulate proliferation of dorsal neuronal populations in the diencephalon and midbrain [24,25]. Of interest, several hedgehog-binding factors (MEGALIN, BOC) and hedgehog effectors (SMO, GLI1, 2, 3, DZIP) are expressed in the developing chicken dorsal midbrain [36]. These expression patterns thus provide a basis for understanding recent reports that implicate hedgehog signaling in the regulation of DMB structures [7,37]. These data, together with the context provided by a novel table of OT histogenetic developmental stages [11], suggest that Shh and Gli may regulate neuronogenesis in the OT by a mechanisms similar to that found in the cerebellum between the Purkinje neurons and the granule neurons; in this case between the efferent macroneurons corresponding to the 2^nd^ neuronal cohort (SGC and SGP neurons) and the associative microneurons derived from the 3^rd^ cohort of neurons (SGFS inteneurons). In fact, by ED2, at the beginning of the DS1, the tectal wall is a single layered neuroepithelium and displays a mNEc density of ≈ 0.4 mNEc/100 μm^2^. Between ED3- ED4, the newborn neurons – the future large efferent neurons of the SGC– can be seen intermingled with the NE cells bodies. By the end of the DS1 (ED4/4.5) the earliest differentiating macroneurons form a PMZ immediately overlying the NEc layer; at this moment a two-fold increase in the mNEc density can be detected at the VZ. Afterwards, between ED4.5 - ED6 (DS3-4), the macroneurons leave the PMZ and the mNEc density decreases abruptly until the end of the proliferating phase.

## Conclusions

Our results indicate that, apart from its critical developmental roles in the VMB patterning, Shh also has a significant influence on cell proliferation and morpho- and histogenesis in the dorsal midbrain. In fact, GliA and Shh electroporations produce several significant changes in the developing OT. Misexpressions of both proteins interfere with the formation of the intertectal fissure located along the dorsal midline and modify the density of mitotic NE cells measured along the DV axis. There is a gradient in the responsiveness of NEcs to the mitogenic effect of Shh and GliA. In fact, this effect is maximal near the dorsal regions and decrease towards the OT-VMB boundary.

In addition, Shh and GliA produce significant changes in the spatial organization of mNEcs. The scaling indexes of the signals derived from the mNEcs records significantly change from values corresponding to stochastic process with poor correlation to highly correlated stochastic process with long memory. The increases observed in the values of β and α reveal that both GliA and Shh install higher correlations or space-dependencies between the values composing the signals representing the mNEc distribution.

Furthermore, the changes in the slope of log-log plots of the number of clusters as a function of the inter-cluster intervals built from the dendrograms obtained by the hierarchical clustering analyses indicate the existence of a power law relationship between “number of clusters” and “inter-cluster interval length” that significantly increases under the GliA and Shh effect.

The extent to which Shh regulates the size of a developing CNS area seems to depend upon the developmental context -developmental stage and position within a spatial reference axis- and the level at which the Hedgehog signaling cascade is perturbed. Our study illustrates that between ED1.5 – ED7 the OT is still growing and malleable and Shh/GliA signaling significantly modifies NE cell proliferation and also influences its spatial organization. These changes, in turn, have profound effects on the OT morphogenesis. Our observations provide a first explanation for the apparent discrepancies found in the literature regarding the role of Shh pathway in midbrain growth. These differences might be due to divergences in the time points chosen for the experiments or the level and extent of ectopic Shh expression/pathway activation. In fact, our gain of function studies does not support a simple morphogen gradient action of Shh. Taken together the findings presented in this paper suggest that the size and shape of the developing midbrain are dependent on dual actions of Shh, influencing both morphogenetic and mitogenic events. Considering the multifunctional uses of the Shh signaling pathway, further understanding of how the levels and duration of hedgehog signaling are modulated by recipient cells is required.

Finally, these findings complement our previous studies in zebra fish and mice and suggest that the mitogenic activity of Shh is likely conserved between teleosts, birds and mammals [4,37].

## Methods

### Chick embryos

Fertilized Leghorn eggs (local farms) were incubated at 38°C with a relative humidity of 90% in a circulated air incubator (G.Q.F. MFG. Co., USA). Embryos were staged according to Hamburger and Hamilton [38].

All animal procedures were in accordance with the Chilean legislation and were approved by Institutional Animal Care and Use Committees.

### Pharmacological manipulation

Treatment included hedgehog inhibitor cyclopamine (Infinity Pharmaceuticals, Inc., Cambridge, MA, USA) and Purmorphamine agonist (Calbiochem, San Diego, CA, USA), both diluted in DMSO vehicle at 10 mM. Eggs were incubated to the appropriate stage and windowed. At E1,5 embryos were injected locally (delivery on top of the OT region) with 0,25 μl of either Cyc or Pur, each drug at a concentration stock of 10 mM. At E5 embryos received an 8 μl application of Cyc or Pur. DMSO was used as vehicle control. The embryos were reincubated until the appropriate stage, verified by morphological criteria focusing on structures such as limbs and heart. For each time window 12–15 embryos per treatment were considered. Three independent rounds of experiments per conditions were performed.

#### Electroporation

For *in ovo* electroporation of select expression constructs, a protocol similar to that described by Agarwala et al. (2001) [8] was followed. Rounds of 10–15 embryos per conditions were performed. Embryos were electroporated with enhanced green fluorescent protein (Green Lantern plasmid, Invitrogen), a dominant active version of human Gli3 high (Gli3A) (kindly donated by Dr E. Briscoe [39]) or Shh (IRES-EGFP-cShh, BD Bioscience Clontech)- containing expression vectors, prepared using a Qiagen plasmid Maxi kit. The injection solution contained 1–3 μg/μl of the selected DNA construct in 10 mM Tris, 1 mM EDTA (pH 7.5) containing 0.1% Fast Green (Sigma) for visual monitoring of the injection. An electro-Square Porator ECM830 electroporation device (BTX, San Diego, CA, USA) was used to generate electric pulses and two gold-plated electrodes, diameter of 0.5 mm and 3 mm length (BTX Model 514), were positioned using micromanipulators such that the resulting current would pass through the OT. Subsequently, a train of square wave pulses was delivered to the embryo with the following parameters: 6 pulses, 25 volts, 50 ms pulse duration, 1 s interpulse interval. Avian Howard’s Ringer's solution containing 10 U penicillin-streptomycin (Sigma) was added to rehydrate the embryo as necessary. Following manipulation the prepared window was sealed with polyethylene tape (Fisher Scientific), and the egg returned to the incubator until harvesting. Electroporated embryos and/or heads of chick embryos were collected. Embryos that showed some sign of injury from electroporation were excluded from further study. Tissue was fixed in 4% paraformaldehyde (PFA) overnight and then processed for staining. Electroporation efficiency was determined by GFP expression *in vivo* and further verified by immunolabeling. Expression of transgenes can be detected by the fluorescence signals from 12–18 h after electroporation onwards. Our analyses were based on whole-mount and on histological sections for in situ hybridization and immunocytochemistry obtained from 60 successfully electroporated embryos with either the Shh (n=34) or GLi3 high (n=26) constructs.

#### Conventional histology

Mitotic NEc records were registered on histological sections of OTs obtained by a standardized histological method. Four specimens were used for each condition (control, GliA and Shh). After fixation, dehydration and embedding in paraffin, midbrains were spatially oriented in order to obtain transverse D-V sections (for details see reference [40]). Sections were rehydrated in a decreasing ethanol gradient and stained with hematoxylin and eosine (H-E), Toluidine Blue or processed for immunohistochemical labeling. Mitotic NEc records were obtained from ED4/4.5 OT –one day after electroporation– since, according to previous studies [11,13,15,16,41] this is the period of highest NEc proliferation rate.

#### In situ hybridization and immunocytochemistry

The whole-mount in situ hybridization was performed according to [39]. Embryos and/or sections were immunolabeled with the primary antibodies listed in Table 1. For whole -mount immunolabeling with Shh, HNF3ß and Pax7 we modified the protocol by [40]. Embryos were fixed in Dent’s fix (4:1 methanol: DMSO) for two hour at room temperature, dehydrated in methanol and left overnight at −20°C for postfix. Before immunostaining embryos were bleached for 24 h at room temperature in Dent’s bleaching (4:1:1; methanol: DMSO: H_2_O_2_). They were rehydrated in PBST (PBS with 1% Triton X-100) and incubated overnight at 4°C with primary antibody diluted in PBST, 5% normal goat serum (NGS) and 5% DMSO. The primary antibodies were washed out six times (1 h each) in TBST (Tris buffer pH 7.5 with 1% Triton) and embryos incubated overnight at 4°C with horse anti-mouse-AP (Vector AP-2000) diluted in TBST with 5% DMSO. Following six washes in TBST, the embryos were incubated for half an hour in AP solution (100 mM Tris pH9.5; 50 mM MgCl2; 100nM NaCl; 1% Tween-20; 2 mM Levamisol) and treated with NBT\BCIP. Embryos were then washed in PBST and incubated overnight with goat anti-rabbit-HRP (Invitrogen) diluted in PBST with 5% DMSO at 4°C. After repeated washes in PBST embryos were incubated finally half hour in DAB and reacted with 0,01% H_2_0_2_. Embryos were postfixed in PFA, washed and stored in 50% glycerol.

**Table 1:** Primary antibodies characteristics

**Antibody/species**	**Supplier/code/lot**	**Immunogen**	**Dilution**
Rabbit polyclonal anti-Notch Homolog 1, Translocation-associated (Notch1)	Lifespan Biosciences	Synthetic peptide CQHSYSSPVDNTPSHQ N-terminal added cysteine Human Notch1 intracell. domain (aa 2488–2502)	1:300
	LS-C16928		
	Lot 6113061		
Rabbit polyclonal anti-neurod (NeuroD1)	Lifespan Biosciences	Synthetic peptide DDDQKPKRRGPKKKKM Conjugated to malemide-activated KLH Human NeuroD1 (aa 76–91)	1:100
	LS-C9245		
	Lot 8030526		
Mouse monoclonal anti-Neuron specific beta III Tubulin [TUJ-1]	Abcam	Rat brain microtubules (whole Protein)	1:500
	ab14545		
	Lot 543886		
Rabbit polyclonal anti-Phospho-Histone H3	Upstate	Linear peptide corresponding to human Histone H3 at Ser10.	1:500
	06-570		
Rabbit polyclonal anti-Green fluorescent protein	Invitrogen	GFP from the jellyfish Aequorea victoria	1:250
	A11122		
Goath polyclonal anti- Patched	Santa Cruz	Peptide mapping at the N-terminus of patched of mouse origin.	1:50
	sc-6149		
Mouse monoclonal anti-PAX7	Hybridoma bank	Recombinant chicken protein made *E*. *coli* (aa 352–523)	1:15
	PAX7		
Rabbit polyclonal anti-sonic hedgehog	Santa Cruz	Rabbit polyclonal anti (aa 41–200) of shh of human origin.	1:50
	sc-9024		
Mouse monoclonal anti-HNF3-**ß**	Hybridoma bank	Recombinant chicken protein made E. coli	1:20
	4C7		

For histological sections preparation, specimens were dehydrated and embedded in paraffin. Ten μm thick serial sections were obtained and collected on gelatinized slides, dried for 1 h at 37°C and then stored at 4°C. Before immunostaining, sections were deparaffinized, rehydrated, rinsed in PBS and processed for antigen retrieval. Nonspecific binding was blocked by preincubating the sections in 5% normal goat serum (NGS) in PBS with 0.05% Triton X-100 (TX-100) for 1 h at RT in humidity chamber. These sections were used for immunolabeling with the antibodies directed to Notch1, NeuroD1, Ptc1, Pax7 and βIIITub, PH3 and GFP.

Immunolabeling was performed with primary antibodies diluted in PBS containing 0.5% NGS (see Table 1). Sections were incubated with the primary antibodies for 20 h at 4°C in humidity chamber. After several rinses in PBS, sections were incubated with secondary antibodies diluted 1:1,000 in PBS for 2 h at RT in a dark humidity chamber. Sections were then rinsed in PBS and counterstained by 10 min incubation with nuclear dye Hoechst 33342 (B-2261, Sigma) in PBS (dilution 1:1,000) at RT in a dark humidity chamber for histoarchitecture analysis. After rinsing, slides were mounted with polyvinyl alcohol mounting medium with DABCO, antifading (10981, Fluka).

Alexa Fluor 488 goat anti-rabbit IgG (H+L) (A-11008, Molecular Probes) and Alexa Fluor 488 F(ab’)2 fragment of goat anti-mouse IgG (H+L) (A-11017, Molecular Probes) were used as secondary antibodies.

##### Antigen retrieval

Antigen retrieval for Notch1 was performed by treatment with 0.1% TX-100 in PBS for 30 min at RT with gentle rotary shaking. βIIITub retrieval was performed by treatment with 0.294% sodium citrate, pH 6 for 15 min at 95°C. NeuroD retrieval with an acidic treatment in 0.1% citric acid, pH 2.7 for 15 min at 95°C was performed in order to reveal NeuroD nuclear reactivity.

##### Controls

Negative controls for the primary antibodies were performed by using several chick adult non-neural and non-endocrine tissues. Negative controls for the secondary antibodies were performed on OT sections of each ED processed without preincubation with primary antibodies. Neither kind of negative control exhibited detectable fluorescence.

#### Mitotic NE cell records

##### Records of mNEc spatial organization

2D maps of mNEc spatial organization were built up by recording the spatial co-ordinates of every mNEc along the OT D-V axis beginning at the dorsal midline. An adaptation of the stereological optical dissector method [42] was used to register mNEcs and fragments of them (For details see reference [11]). Capture of histological images and data recording were performed with an image-processing device (Carl Zeiss, Oberkochen, Germany) consisting of an Axioplan 2 imaging optical epifluorescence microscope with an Axiocam HR color digital scanner connected to a computer equipped with several software.

Mitotic NEc records were performed according to the following protocol:

Complete series of partial digital images (400X) obtained along the entire ILM of each section were orderly assembled to obtain a single *complete image* of the ILM along the D-V section. Every mNEc recognized in the “*Live*” window [1000X (final digital amplification: 5000X)] was identified in the *complete image* and recorded with the “*events*” tool of the Axiovision software. Sets of data corresponding to different sections were saved as *.csv files (csv: comma-separated values). These files contain five sets of data: 1) Record number: indicates the serial number (1^st^, 2^nd^ … n^th^ position) of each mNEc taken as reference “0” a label located at the dorsal midline; 2) Magnitude: indicates which variables are evaluated (count, co-ordinate, etc.); 3) Unit: indicates the magnitude unit (number, mm, etc.); 4 and 5) Values 1 and 2: specify the co-ordinates (***x***,***y***) of every mNEc within the OT complete image taking as co-ordinate origins (0,0) the left uppermost pixel of the complete image.

**Figure 14:**
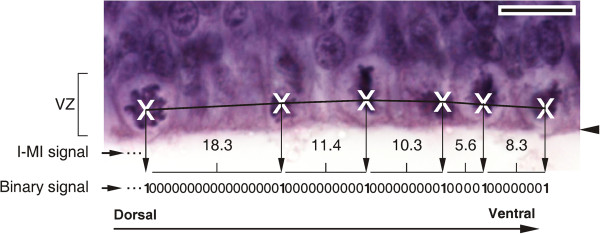
**Mitotic neuroepithelial cell recording and signal generation.** Image of the OT ventricular zone illustrating a segment of the mNEc-R (white crosses). Corresponding subseries of the I-MI signal (Euclidian distances between successive mitosis) and of the binary signal (sequence of “*1 s*” and “*0 s*”) derived from the mNEc-R are also shown. The Arabic numbers between successive crosses indicates the I-MI length expressed in μm. The vertical arrows indicate the spatial correlation between the mNEcs positions and the positions of “*1 s*” in the binary signal. The horizontal lines between successive arrows indicate the distance (number of “*0 s*” + 1) between successive “*1 s*” of the binary signal. Horizontal arrowhead: ILM. VZ: ventricular zone. Bar: 10 μm.

The spatial distribution of mNEcs was recorded on D-V histological sections performed halfway between the cephalic and the caudal poles of the OT. Ten mNEc records (5 right and 5 left hemispheres) were obtained from each OT. Three different signals representative of the proliferation activity were derived from each mNEc record (Figure 14).

(a) *Inter*-*mitotic Interva l*(*I*-*MI*) *signal*. I-MI signals were calculated as the Euclidian distances between the centers of two successive mNEcs from the dorsal to the ventral midline.

(b) *Binary* [*0*–*1*] *signals*. Represent the mNEc distribution as a model of a stochastic point process. The sampling interval was 1 μm length. Each *1* (positive event) correspond to the center of a mNEc. The space occupied by interphasic NEc (absence of event) was represented as subseries of *0 s*. The number of *0 s* between two adjacents *1 s* corresponds to the I-MI length.

(c) *mNEc density signal*. Reveals the variability in mNEc density (number of mNEc/100 μm^2^) measured in successive spatial windows from the dorsal to the ventral midline. The mean value of the I-MI length was used as spatial windows. The expression [n/wl (μm) * sth (μm)] * 100 [where: n = number of mNEcs, wl = window length and sth = section thickness] was used to estimate the mNEc density.

#### Signal analyses

The following algorithms, implemented in Matlab™ software (The MathWorks, Inc. MATLAB, Natick, Massachusetts), were used to estimate the scaling indexes of the signals representing the mNEc spatial organization.

I-MI signals and mNEc density signals were analyzed by means of the classical spectral analysis or power spectral density (PSD), i.e. the Fourier Transform of the autocorrelation function. The PSD allows estimating the exponent β and to differentiate non-correlated (white noise) processes from 1/f processes with dependency and memory.

Binary signals were analyzed by three different algorithms: (a) the Fano Factor allows estimating the exponent α characteristic of stochastic point processes and to differentiate uncorrelated homogeneous Poisson process from correlated fractal processes; (b) the Box Counting method estimates the fractal dimension (space filling property) and allows to differentiate amongst different patterns of spatial distribution; (c) the hierarchical clustering analysis (HCA) allows detecting a hierarchical organization characterized by power law relationship between the number of clusters and the inter-cluster intervals length. This analysis allows characterizing fractal organizations (self-similarity or clusters within clusters) that cannot be defined by a typical mean cluster size or by a typical mean inter-cluster interval length.

All these non-linear methods have proven efficacy as mathematical tools to analyze and characterize which kind of stochastic process an empirical signal fits [17-21].

#### Statistical analyses

Several kinds of comparisons amongst sets of data [a) percentage of the ILM area occupied by mNEcs; b) fractal dimenssion or space filling property, c) I-MI length, d) mNEcs density and e) scaling exponents] corresponding to controls and Shh and Gli electroporated midbrains were processed statistically by means of the Statistics Toolbox™. Statistical significance of the differences was determined by the one-way analysis of variance (ANOVA) followed by multiple comparisons of means using Tukey-Kramer post-hoc test. A value of p<0.05 was considered as statistically significant.

## Abbreviations

βIIITub: Beta III Tubulin; CNS: Central nervous system; Cyc: Cyclopamine; DMB: Dorsal Midbrain; D-V: Dorsal-ventral; DS: Developmental stage; FF: Fano Factor; GliA: Gli activator; HCA: Hierarchical clustering analyses; H-E: Hematoxylin and eosine; Hnf3β: Hepatocyte nuclear factor 3-beta; ILM: Inner limiting membrane; I-MI: Inter-mitotic intervals; mNEc: Mitotic NEc; NEc: Neuroepithelial cells; OT: Optic tectum; Pax7: Paired box homeotic gene 7; PH3: Phospho-histone H3; PMZ: Premigratory zone; PSD: Power Spectral Density; Ptc1: Patched1; Pur: Purmorphamine; SGC: Stratum Griseum Centrale; SGFS: Stratum Griseum et Fibrosum Superficiale; SGP: Stratum Griseum Periventriculare; Shh: Sonic hedgehog; VMB: Ventral Midbrain; VZ: Ventricular zone.

## Authors’ contributions

GC and ME performed electroporation procedures. JB and ME accomplished the pharmacological treatments and made the PH3, Pax7, Ptc1, Shh and GFP immunohistochemical studies. MR and SD performed mNEc records, statistical analyses and Notch, βIII-tub and NeuroD immunohistochemical procedures. MR participated in the design of the study and performed non-linear analyses. VP and VF conceived the study. VP designed and coordinated the electroporation and immunohistochemical procedures and wrote the manuscript. VF designed and coordinated the theoretical analyses and wrote the manuscript. All authors read and approved the final manuscript.
